# Contrasting the Role of Pores on the Stress State Dependent Fracture Behavior of Additively Manufactured Low and High Ductility Metals

**DOI:** 10.3390/ma14133657

**Published:** 2021-06-30

**Authors:** Alexander E. Wilson-Heid, Erik T. Furton, Allison M. Beese

**Affiliations:** 1 Department of Materials Science and Engineering, Pennsylvania State University, University Park, PA 16802, USA; axw66@psu.edu (A.E.W.-H.); emf5640@psu.edu (E.T.F.); 2 Department of Mechanical Engineering, Pennsylvania State University, University Park, PA 16802, USA

**Keywords:** ductile fracture, stress state, Ti-6Al-4V, 316L stainless steel, laser powder bed fusion

## Abstract

This study investigates the disparate impact of internal pores on the fracture behavior of two metal alloys fabricated via laser powder bed fusion (L-PBF) additive manufacturing (AM)—316L stainless steel and Ti-6Al-4V. Data from mechanical tests over a range of stress states for dense samples and those with intentionally introduced penny-shaped pores of various diameters were used to contrast the combined impact of pore size and stress state on the fracture behavior of these two materials. The fracture data were used to calibrate and compare multiple fracture models (Mohr-Coulomb, Hosford-Coulomb, and maximum stress criteria), with results compared in equivalent stress (versus stress triaxiality and Lode angle) space, as well as in their conversions to equivalent strain space. For L-PBF 316L, the strain-based fracture models captured the stress state dependent failure behavior up to the largest pore size studied (2400 µm diameter, 16% cross-sectional area of gauge region), while for L-PBF Ti-6Al-4V, the stress-based fracture models better captured the change in failure behavior with pore size up to the largest pore size studied. This difference can be attributed to the relatively high ductility of 316L stainless steel, for which all samples underwent significant plastic deformation prior to failure, contrasted with the relatively low ductility of Ti-6Al-4V, for which, with increasing pore size, the displacement to failure was dominated by elastic deformation.

## 1. Introduction

A major area of focus in the field of additive manufacturing (AM) is understanding pore formation, and process optimization with the goal of creating fully dense, defect-free components [1,2,3]. Studying the effect of pores on metal failure, along with the development of failure models based on theory and experiments has a long and continued history for conventionally processed ductile metals due to the importance of avoiding failure in load bearing components or during metal forming [4,5,6,7,8,9]. For example, in 1977 Gurson developed a model in terms of a yield function and microstructurally informed void volume fraction to understand void growth and ductile failure using simplified spherical and cylindrical void models [6]. By combining the existing frameworks for fracture modeling with the unique manufacturing capabilities of AM, new insight into the effect of pores on ductile failure is possible.

Laser powder bed fusion (L-PBF) AM is a process for building metallic components layer-by-layer using a focused laser heat source to melt a selected 2-dimensional pattern in a thin layer (10–100 µm) of powder feedstock to a baseplate, lowering the baseplate by a prescribed layer thickness, spreading on a new layer, scanning the next layer pattern, which fuses to the previous layer, and repeating until a final 3-dimensional (3D) component is completed. In AM, there are numerous processing parameters that dictate a completed component’s quality. One primary evaluation metric for optimizing a parameter set for a given alloy is component density, or the reduction of pores. Pores in AM parts can be formed via different mechanisms [10]; two of the primary mechanisms are: (1) gas entrapment during melting and solidification of the meltpools, analogous to that seen in casting and welding, which generates spherical pores and (2) irregular morphology lack-of-fusion (LoF) pores, which are formed due to incomplete fusion between layers or adjacent meltpools on the same layer along the heat source scanning path, and which can have sharp features. LoF pores are of primary interest in AM because of their significantly more detrimental influence on material ductility compared to spherical pores [11].

In addition to pores having a harmful effect on the fracture behavior of ductile metals, stress state is also known to play an important role in fracture [12,13,14]. Stress state can be defined using the two parameters stress triaxiality (*η*) and Lode angle parameter ($\bar{\theta }$). The stress triaxiality is the ratio of the mean stress ($\sigma_{m}$) and von Mises equivalent stress (${\bar{\sigma }}_{VM}$):

(1) $$\eta =\frac{\sigma_{m}}{{\bar{\sigma }}_{VM}}\;\mathrm{with}\;\sigma_{m}=\frac{1}{3}I_{1}\;\mathrm{and}\;{\bar{\sigma }}_{VM}=\sqrt{3J_{2}}$$

where *I*_1_ = *σ_kk_* is the first invariant of the stress tensor, σ, and *J*_2_$=\frac{1}{2}s_{ij}s_{ij}$ is the second invariant of the deviatoric stress tensor, ***s***. The normalized Lode angle parameter is a function of the third invariant of the deviatoric stress tensor, *J*_3_$=det\left(s_{ij}\right)$, and is defined as:

(2) $$\bar{\theta }=1-\frac{2}{\pi }arccos\left[\frac{27}{2}\frac{J_{3}}{{\bar{\sigma }}_{VM}^{3}}\right].$$

Increased stress triaxiality is known to accelerate the void nucleation and growth process in ductile metals, resulting in reduced ductility even in fully dense samples [5,15]. Designing load bearing components to be safe under the spatially varying stress state is an important consideration for engineers in the design against failure.

**Figure 1:**
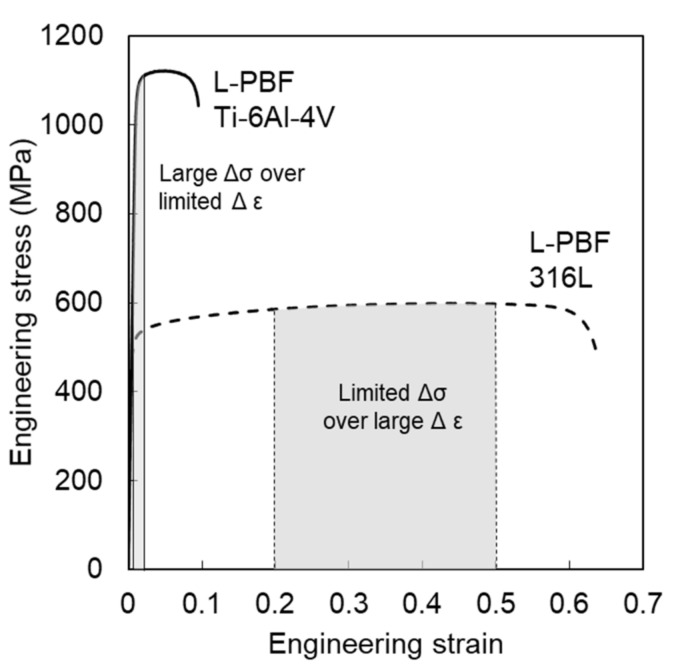
Uniaxial tension engineering stress vs. strain curves for L-PBF Ti-Al-4V and 316L that schematically highlights the differences of resolution in stress and strain for the two alloys.

Fracture models that describe the effects of stress state on ductile failure have primarily been presented in mixed stress-strain space, meaning equivalent plastic strain to failure versus *η* and $\bar{\theta }$ (referred to here as equivalent strain space) because in ductile fracture, the resolution in strain is typically much larger than the resolution in stress, that is, large differences in strain result in relatively minor differences in stress due to the fact that the elastic contribution to failure is negligible compared to the plastic contribution. However, for alloys with little to no plastic deformation to failure, describing failure in terms of strain to failure becomes more challenging. This is shown schematically in Figure 1, which compares the engineering stress-strain curves for the two materials in this study—Ti-6Al-4V and 316L stainless steel (316L).

In this study, the effect of pores relative to the behavior of dense samples was assessed in two different alloys using well-known fracture models calibrated in both equivalent stress and strain space. Data over a wide range of stress states from previous studies by the authors on L-PBF stainless steel 316L [16,17] and L-PBF Ti-6Al-4V [18,19,20] that included intentionally manufactured, penny-shaped pores of varying diameter were used to calibrate fracture models for each pore size in stress triaxiality versus Lode angle parameter versus equivalent stress space (Haigh-Westergaard space, referred to here as equivalent stress space) and equivalent strain space. By comparing the ductile 316L alloy (>60% engineering strain to failure under uniaxial tension) to the less ductile Ti-6Al-4V (<10% engineering strain to failure under uniaxial tension) in both equivalent stress and strain space as a function of pore size, an assessment of dominant fracture mechanism changes, if any, can be made for each material. Additionally, the appropriateness of fracture models in equivalent stress space versus equivalent plastic strain space for capturing the effect of pore size on the failure behavior of both materials is discussed. 

## 2. Materials and Methods

The complete description of the manufacturing process, samples, experimental set-up, and simulations are described in extensive detail for the 316L builds in Refs. [16,17] and for the Ti-6Al-4V builds in Refs. [18,20]. The following is an overview of information that is most pertinent to the current study. 

### 2.1. Experimental Methods

**Figure 2:**
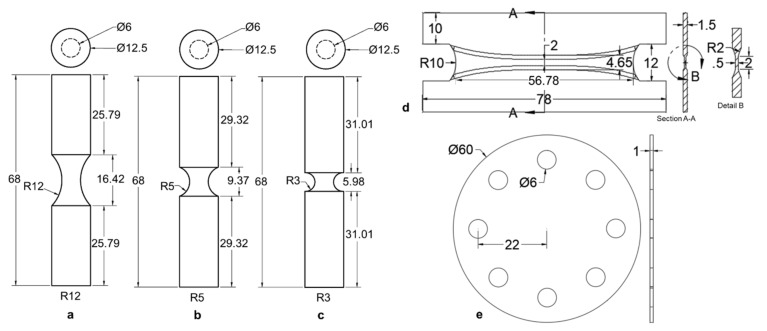
Drawings of mechanical test specimens used to calibrate fracture criterion. (**a**–**c**) The cylindrical notched tension geometries that were tested in the dense condition and with single, penny-shaped pores of varying diameter at the center. (**d**) The butterfly test geometry used to evaluate material properties under pure shear and (**e**) the punch test geometry used to evaluate equibiaxial tension. Both (**d**,**e**) were only tested in the dense condition. All dimensions in mm.

Samples used in this study for both alloys were manufactured on a 3DSystems, Inc. ProX 320 L-PBF AM machine (3DSystems, Rock Hill, SC, USA). No post-processing heat treatment was used for the 316L builds, while a post-processing stress relief heat treatment of 650 °C for 3 h in an argon environment was used for the Ti-6Al-4V builds. Data obtained using five different sample geometries, selected as they have a pre-determined failure initiation location, and corresponding to five unique stress states, were used in the current study: pure shear, equibiaxial tension (punch tests), and round notched tension with three different notch radii (3 mm (R3), 5 mm (R5), and 12 mm (R12)), as shown in Figure 2. The three notched tension geometries each had a minimum cross-sectional diameter of 6 mm. The gauge regions of all samples were fabricated using computer numeric control (CNC) machining (Lynx 220L, Doosan Machine Tools Co., Ltd., Pine Brook, NJ, USA).

The five sample geometries for both materials were evaluated in the dense condition, that is using process parameters optimized to build each material. Only the R3, R5, and R12 tests selected in the current study had single, penny shaped pores of varying diameters that were directly designed into the CAD files for the samples, and therefore, included at the center of each sample during the AM fabrication. The pore diameters in µm (and their percentage of the cross-sectional area of the notched tension samples) evaluated in this study were: 300 (0.3%), 600 (1%), 1200 (4%), and 2400 (16%). The four pore sizes were chosen in the current analysis because they interrogated a wide range of percentage of the cross-sectional area that led to varied mechanical response relative to dense samples; additionally, all four pore sizes were studied in each alloy, allowing for a direct comparison. In the 316L material, each of the pores were designed to be 180 µm or 3 layers tall cylinders with the varying diameters, while in the Ti-6Al-4V samples, the internal pores were designed with a conical feature on the top surface after 180 µm vertical walls to prevent pore closure due to dross formation in this alloy system. As discussed in Ref. [20], a study on the effect of pore height performed for Ti-6Al-4V indicated that the pore height did not impact the measured strength or ductility in uniaxial tension specimens.

For all tests at least two repetitions were completed for each combination of stress state and pore size, including the dense samples. Tests were performed to failure on their respective load frames. Displacement and strain, using a virtual extensometer, were measured using digital image correlation (DIC), a non-contact surface strain field measurement technique. For each test, two different definitions of failure were examined, displacement to maximum force and displacement to material separation, and were used to inform the simulation data in Section 2.1 and for calibration of the equivalent stress and strain fracture models, respectively, in Section 2.3. 

### 2.2. Finite Element Analysis Simulations

Calibrated plasticity models for 316L [17] and Ti-6Al-4V [19] were developed previously by the authors and used in simulations of each dense geometry using the finite element method in the commercial software Abaqus [21]; the complete model details for each fracture geometry are provided in Refs. [16,17,18,20]. Finite element analysis (FEA) simulations of each dense geometry were used to probe, as a function of applied displacement, the components of the Cauchy stress tensor (σ), stress state (*η* and $\bar{\theta }$), and von Mises equivalent stress (${\bar{\sigma }}_{VM}$) and equivalent plastic strain ($\bar{\varepsilon }$) in the centermost element of each sample geometry, which is where failure is assumed to initiate. Simulations were performed to mimic the experimental displacement to catastrophic failure of the dense specimens, as measured via the virtual extensometer in experiments and an equivalent extensometer in each of the FEA models. The average stress triaxiality and Lode angle parameter up to the experimentally measured displacement to failure for each test condition (i.e., pore size and sample geometry) were calculated using:

(3) $$\eta_{av}=\frac{1}{{\bar{\varepsilon }}_{f}^{p}}\int_{0}^{{\bar{\varepsilon }}_{f}^{p}}\eta \;d\bar{\varepsilon }$$

and:

(4) $${\bar{\theta }}_{av}=\frac{1}{{\bar{\varepsilon }}_{f}^{p}}\int_{0}^{{\bar{\varepsilon }}_{f}^{p}}\bar{\theta }\;d\bar{\varepsilon }.$$

The equivalent stress from the FEA model was recorded at the displacement corresponding to the average experimental displacement to maximum force for every test condition. The equivalent plastic strain from the FEA model was recorded at the displacement corresponding to the average experimental displacement to catastrophic failure, or material separation, for every test condition. 

To summarize, for both materials the data sets were analyzed in two different 3D spaces ($\eta_{av}$, ${\bar{\theta }}_{av}$, ${\bar{\sigma }}_{VM}$), or equivalent stress space, and ($\eta \left(\bar{\varepsilon }\right)$, $\bar{\theta }\left(\bar{\varepsilon }\right)$, ${\bar{\varepsilon }}_{f}$), or equivalent strain space. For each 3D space there were five data points for the dense condition (pure shear, punch, R3, R5, and R12) and three additional data points for each pore size (R3, R5, and R12). These data will be referred to in subsequent sections in the calibration of the fracture models in equivalent stress and strain spaces. 

### 2.3. Fracture Models

In this study, three fracture models were calibrated in the 3D equivalent stress space of ($\eta_{av}$, ${\bar{\theta }}_{av}$, ${\bar{\sigma }}_{VM}$) and two fracture models in the 3D equivalent strain space of ($\eta \left(\bar{\varepsilon }\right)$, $\bar{\theta }\left(\bar{\varepsilon }\right)$, ${\bar{\varepsilon }}_{f}$), which are transformed versions of two of the stress-based models using assumed plasticity frameworks as described in Refs. [22,23]. Each model was calibrated for the dense material and each pore size. For the fracture surface calibrations for dense material, the dense pure shear, punch, R3, R5, and R12 data were used. For each subsequent pore size, five tests were also used in calibration: the dense pure shear and punch data were used as “anchor points” as the pore configurations being considered in this study (with the cylinder axis oriented parallel to the vertical build direction) are assumed to have relatively negligible impact on ductility in those stress states, and the three notched tension data for the corresponding pore size. 

#### 2.3.1. Equivalent Stress versus Stress Triaxiality and Lode Angle Space

##### Maximum Stress Failure Criterion

The maximum principal stress fracture criterion is based on the premise that a crack or defect will grow in a direction perpendicular to the maximum principal stress, resulting in failure when the maximum principal stress reaches a critical value. This model is given as:

(5) $$\sigma_{c}=\sigma_{max}=\max\left(\sigma_{1},\;\sigma_{2},\;\sigma_{3}\right)$$

where $\sigma_{1}$, $\sigma_{2}$, and $\sigma_{3}$ are the principal stress components, and $\sigma_{c}$ is the critical maximum principal stress resulting in fracture. For each notched tension geometry, the average $\sigma_{c}$ values from the three notched tensions tests for the dense data, as well as each pore size, were calculated and are given in Table 1 and Table 2 for both materials. To plot the maximum stress fracture loci for dense material and each pore size, von Mises equivalent stress, *η*, and $\bar{\theta }$ were calculated and plotted as a function of the three principal stress components with $\sigma_{c}$ held constant. The discrete points were then interpolated to generate a fracture locus using only a single input, $\sigma_{c}$.

##### Mohr-Coulomb Failure Criterion

The Mohr-Coulomb (MC) fracture criterion is a classical stress-based criterion that has its origins in describing brittle failure (e.g., rocks and ceramics) [24]. The model is phenomenological in that it describes fracture as occurring when a combination of normal stress (the intermediate principal stress is ignored) and shear stress reach a critical value. The model in the 3D space of (η, $\bar{\theta }$, ${\bar{\sigma }}_{VM}$) was initially presented by Bai et al. [22] and is given as:

(6) $${\bar{\sigma }}_{vM}^{MC}\left[\eta ,\bar{\theta }\right]=c_{2}{\left[\sqrt{\frac{1+c_{1}^{2}}{3}}cos\left(\frac{\pi }{6}-\bar{\theta }\right)+c_{1}\left(\eta +\frac{1}{3}sin\left(\frac{\pi }{6}-\bar{\theta }\right)\right)\right]}^{-1}$$

where $c_{1}$ (friction coefficient) and $c_{2}$ (shear resistance) are model parameters that are calibrated. The ranges of the model parameters are $c_{1}\geq 0$ and $c_{2}>0$. The optimized model parameters for the dense material and the different pores sizes were determined using a Matlab function (fmincon) (R2021a, 2021, Mathworks, Natick, MA, USA) that finds the minimum of a constrained nonlinear multivariable function; in the current study, this function probed fracture model parameters in a defined range and calculated the mean absolute percentage error (MAPE) between the predicted equivalent stress on the fracture surface for each evaluated parameter set and the fixed combined experimental/simulation scatter data at the same (η, $\bar{\theta }$) coordinates. The optimized parameter sets for each pore size are shown in Table 1 and Table 2.

##### Hosford-Coulomb Failure Criterion

The Hosford-Coulomb (HC) model is a stress-based failure criterion that describes failure in terms of a material’s deviatoric strength, as originally postulated by Coulomb in 1776 [25]. Unlike the MC model, the HC model considers the contribution from the intermediate principal stress by replacing the maximum shear stress contribution in the MC model with the Hosford equivalent stress [26]. The model in the 3D space of (η, $\bar{\theta }$, ${\bar{\sigma }}_{VM}$) was initially presented by Mohr and Marcadet [14] and is given as:

(7) $${\bar{\sigma }}_{vM}^{HC}\left[\eta ,\bar{\theta }\right]=\frac{b}{{\left\{\frac{1}{2}\left[{\left(f_{1}-f_{2}\right)}^{a}+{\left(f_{2}-f_{3}\right)}^{a}+{\left(f_{1}-f_{3}\right)}^{a}\right]\right\}}^{\frac{1}{a}}+c\left(2\eta +f_{1}+f_{3}\right)}$$

with:

(8) $$f_{1}=\frac{2}{3}\cos\left[\frac{\pi }{6}\left(1-\bar{\theta }\right)\right]\;f_{2}=\frac{2}{3}\cos\left[\frac{\pi }{6}\left(3+\bar{\theta }\right)\right]\;f_{3}=-\frac{2}{3}\cos\left[\frac{\pi }{6}\left(1+\bar{\theta }\right)\right]$$

where model parameters *a* (Hosford exponent—controls the Lode angle parameter dependence), *b* (controls the height of the fracture surface), and *c* (controls the stress triaxiality dependence) were calibrated for each pore size in the current study. The Matlab function (fmincon) used in the calibration of the MC model parameters was adopted for the HC optimization using the same experimental/simulation data for each pore size. The optimized parameter sets for each pore size are shown in Table 1 and Table 2.

#### 2.3.2. Equivalent Plastic Strain versus Stress Triaxiality and Lode Angle Space

Taking into account the higher resolution in strain to failure than stress to failure generally observed in ductile metals, the stress-based fracture criteria can be transformed to strain space through a transformation based on an appropriate plasticity model framework as described in Refs. [14,22]. For both materials discussed here, isotropic plasticity models were assumed and used in simulations. 

The first strain-based fracture model investigated was the modified Mohr-Coulomb (MMC) fracture criterion [22]. This model is based on transforming the MC model from stress space to strain space by assuming a plasticity framework as described in [22]. This results in a definition of strain to failure, under the constraints of proportional loading, in ($\eta_{av}$, ${\bar{\theta }}_{av}$, ${\bar{\varepsilon }}_{f}$) space of:

(9) $$\begin{array}{ll} {\bar{\varepsilon }}_{f}^{MMC}=\{\frac{A}{c_{2}}[c_{\theta }^{s} & +\frac{\sqrt{3}}{2-\sqrt{3}}\left(c_{\theta }^{ax}-c_{\theta }^{s}\right)\left(sec\left(\frac{\bar{\theta }\pi }{6}\right)-1\right)][\sqrt{\frac{1+c_{1}^{2}}{3}}cos\left(\frac{\bar{\theta }\pi }{6}\right)\\ & +c_{1}\left(\eta +\frac{1}{3}sin\left(\frac{\bar{\theta }\pi }{6}\right)\right)]{\}}^{-\frac{1}{n}} \end{array}$$

with:

(10) $$c_{\theta }^{ax}=\left\{\begin{array}{ll} 1 & \bar{\theta }\geq 0\\ c_{\theta }^{c} & \bar{\theta }<0 \end{array}\right.\;.$$

The parameters *A* and *n* are parameters from the equations used to describe the rate of strain hardening in the plasticity models given in [17,19], and these values were held constant for each pore size, while $c_{1}$, $c_{2}$, $c_{\theta }^{s}$, and $c_{\theta }^{c}$ were calibrated for each pore size in the current study. The parameters $c_{1}$ and $c_{2}$ have the same effect as in the stress-based MC model, and $c_{\theta }^{s}$ and $c_{\theta }^{c}$ control the Lode angle parameter dependence and asymmetry, respectively, of the calibrated fracture surfaces.

The Hosford-Coulomb fracture criteria in equivalent plastic strain space is a phenomenological fracture model that was developed on the hypothesis that the fracture initiation in a ductile metal coincides with the formation of a primary or secondary band of localization, where the moment of this localization can be described by a critical combination of the Hosford equivalent stress and the normal stress on the plane of maximum shear. Marcadet and Mohr [14] and Gu and Mohr proposed strain-based fracture models based on this critical combination of equivalent and normal stress by transforming the stress-based failure criterion to strain space through assumed plasticity models. The model used here is the one proposed in Gu and Mohr [23], which, under the constraints of proportional loading, is given as:

(11) $${\bar{\varepsilon }}_{f}^{HC}\left(\sigma /\bar{\sigma }\right)=b{\left(\frac{1+c}{g_{HC}\left(\frac{\sigma }{\bar{\sigma }}\right)}\right)}^{\frac{1}{d}}$$

with:

(12) $$g_{HC}\left(\frac{\sigma }{\bar{\sigma }}\right)={\left\{\frac{1}{2}\left[{\left(f_{1}-f_{2}\right)}^{a}+{\left(f_{2}-f_{3}\right)}^{a}+{\left(f_{1}-f_{3}\right)}^{a}\right]\right\}}^{\frac{1}{a}}+c\left(2\eta +f_{1}+f_{3}\right).$$

In this model, *a*, *b*, and *c* all retain their meaning from the HC stress-based failure criterion and *d* increases or decreases the curvature of the fracture locus, where a larger value of *d* results in less curvature and therefore a flatter surface. 

Parameters for both models were calibrated using a Matlab code (fmincon) that optimized parameter values such that mean absolute percentage error for the damage indicator (D), calculated as:

(13) $$D=\int_{0}^{{\bar{\varepsilon }}_{f}}\frac{1}{{\bar{\varepsilon }}_{f}^{MMC,}}\;d\bar{\varepsilon }\;\mathrm{and}\;D=\int_{0}^{{\bar{\varepsilon }}_{f}}\frac{1}{{\bar{\varepsilon }}_{f}^{HC}}\;d\bar{\varepsilon },$$

was minimized. A target value of D = 1, corresponding to material failure, was used in the optimization code for calculating error.

## 3. Results and Discussion

### 3.1. Effect of Pores in Equivalent Strain versus Stress Triaxiality and Lode Angle Parameter Space

**Figure 3:**
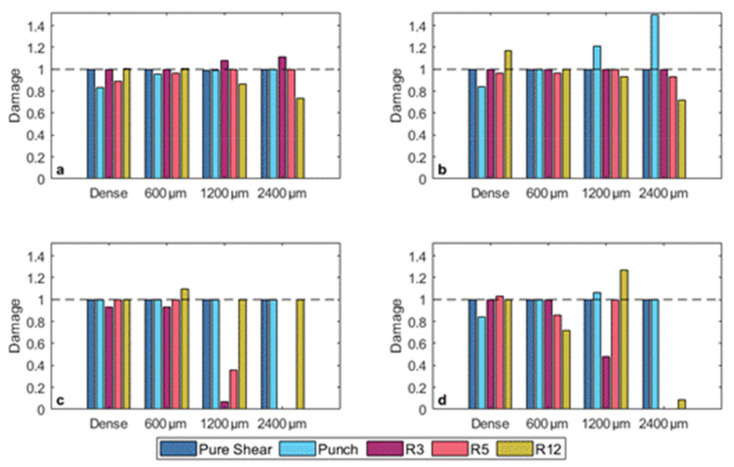
Damage accumulation at fracture for (**a**,**b**) 316L and (**c**,**d**) Ti-6Al-4V using the calibrated (**a**,**c**) modified Mohr-Coulomb and (**b**,**d**) Hosford-Coulomb models. The dashed line at a value of damage equal to one represents perfect agreement between the model and the experimental/simulation data for each test. Numbers on the horizontal axes denote the pore diameter at the center of the samples.

The results from the models in the equivalent plastic strain space will first be discussed because these models are most often used when describing ductile failure behavior and provided a baseline performance with which the equivalent stress-based models were contrasted. The results of the strain-based fracture model calibration for both the MMC and strain-based HC model are shown in Figure 3 and the resulting model parameters are given in Table 3 and Table 4.

#### 3.1.1. L-PBF 316L

**Figure 4:**
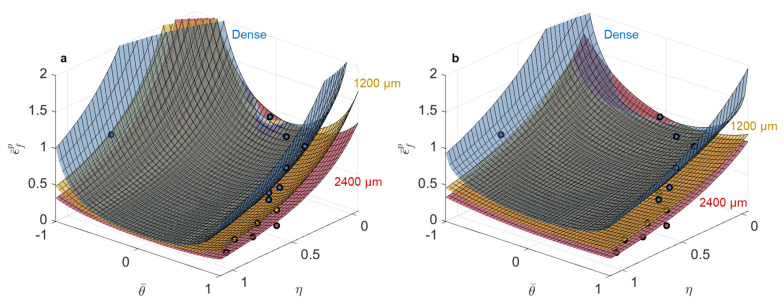
Calibrated (**a**) modified Mohr–Coulomb and (**b**) Hosford–Coulomb fracture loci in stress triaxiality vs. Lode angle parameter vs. equivalent plastic strain space for 316L using the dense, 1200 µm, and 2400 µm pore data. Note that the horizontal positions of each symbol correspond to the average stress triaxiality and Lode angle parameter during loading, while the vertical position corresponds to the experimental strain to failure of the average stress state under the assumption of proportional loading; therefore, the symbols are not expected to lie on the fracture surfaces, which took into account loading history in the accumulation of damage.

The damage prediction shows that for 316L, both the MMC and strain-based HC models do a relatively good job at accurately capturing the failure behavior of the dense samples and the samples with 600 µm and 1200 µm diameter intentional pores. The mean absolute percentage error across all test conditions was 4.7% for the MMC model and 6.7% for the HC model. However, for the HC model the largest error was for the 2400 µm pore diameter (16% of the cross-sectional area), where the model did not accurately capture the punch, R5, and R12 behavior simultaneously, resulting in a MAPE of 17%. The low error in the 316L calibrated parameters resulted in good fitting of the fracture surfaces relative to the experimental/simulated scatter data, as shown in Figure 4.

With increased pore size in the 316L, there was a noticeable flattening behavior of the surfaces using both the MMC and strain-based HC models; in other words, failure behavior became less stress state dependent as a function of increased pore size. The strain-based models captured the fracture behavior in 316L with the inclusion of pores well because even with the introduction of the 2400 µm diameter pore (16% of the cross-sectional area), the equivalent plastic strain to failure was >20% at failure, which significantly exceeds elastic deformation; thus, these data still lie in the region of high strain resolution. Overall, the MMC model did a better job capturing the fracture behavior as a function of pore size using the five tests in this study for calibration of the model parameters.

#### 3.1.2. L-PBF Ti-6Al-4V

The calibrated model parameters for Ti-6Al-4V had relatively low average error in the damage prediction for the dense, 300 µm, and 600 µm diameter pore experimental/simulation test data using both the calibrated MMC (5.2% MAPE) and the strain-based HC (4.5% MAPE) models. However, the ability of the models to accurately describe the evolution of damage to material failure drastically declined for the samples with the 1200 µm diameter (4% of the cross-sectional area) and 2400 µm diameter (16% of the cross-sectional area) pores. These large errors are primarily driven by errors in fitting the notched tension tests, where the inclusion of the large diameter pores results in failure at little to no plastic strain [20]. 

**Figure 5:**
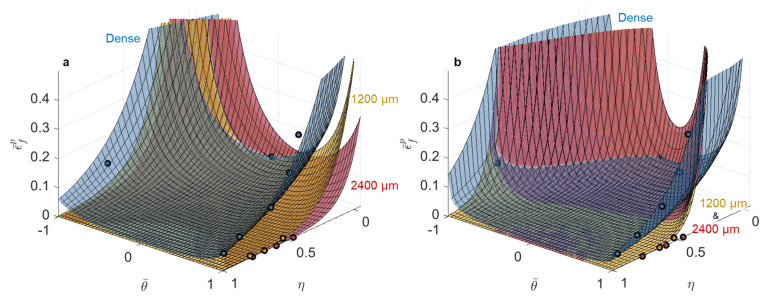
Calibrated (**a**) modified Mohr–Coulomb and (**b**) Hosford–Coulomb fracture loci in stress triaxiality vs. Lode angle parameter vs. equivalent plastic strain space for Ti-6Al-4V using dense, 1200 µm, and 2400 µm pore data. Note that the horizontal positions of each symbol correspond to the average stress triaxiality and Lode angle parameter during loading, while the vertical position corresponds to the experimental strain to failure of the average stress state under the assumption of proportional loading; therefore the symbols are not expected to lie on the fracture surfaces, which took into account loading history in the accumulation of damage.

Plotting the fracture surfaces for 1200 µm diameter (4% of the cross-sectional area) and 2400 µm diameter (16% of the cross-sectional area) pores, as shown in Figure 5 for both models, resulted in surfaces that were hard to distinguish from each other and did not perfectly capture the data. The breakdown of the models’ ability to capture the data accurately in the equivalent plastic strain space for the test conditions with the largest pores was due to the fact that, with increased pore size and reduced displacement to failure, the elastic strain contribution became non-negligible or even dominant, compared to the plastic contribution, for failure in the Ti-6Al-4V.

### 3.2. Effect of Pores in Equivalent Stress versus Stress Triaxiality and Lode Angle Parameter Space

#### 3.2.1. L-PBF 316L

The equivalent strain-based models captured the change in fracture behavior with increasing pore diameters well, and based on the limited strain hardening in the 316L as shown in Figure 1, the ability to resolve the change in equivalent failure stress, with increasing pore size, should be restricted. Both the MC and HC stress-based models captured the experimental/simulated data well with all calculated MAPE below 10%. 

**Figure 6:**
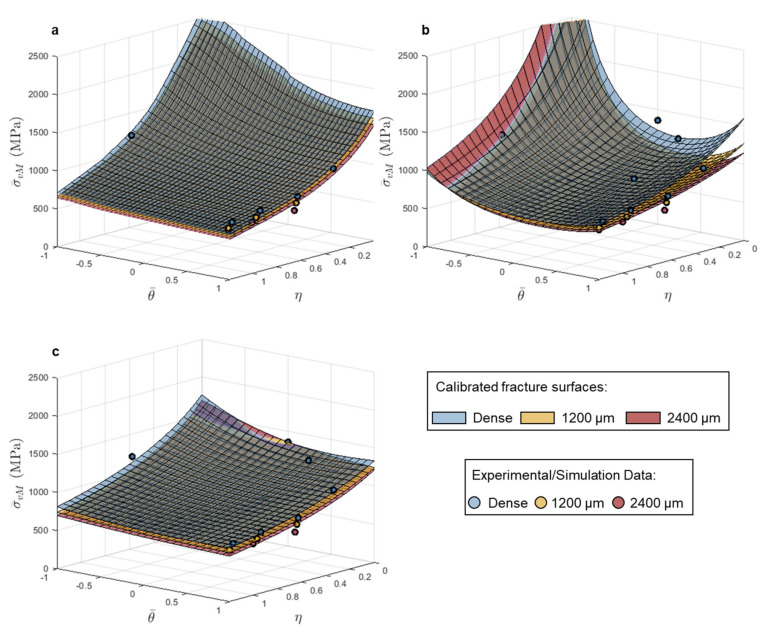
Calibrated (**a**) maximum stress, (**b**) Mohr–Coulomb and (**c**) Hosford–Coulomb fracture loci in stress triaxiality vs. Lode angle parameter vs. equivalent stress space for 316L using dense, 1200 µm, and 2400 µm pore data.

The fracture surface shapes remained similar with increasing pore size, but the magnitude of equivalent stress across the entire surface was reduced as pore size increased. The MC model exhibits the most change in shape with increased pore size, where the calibrated models for the samples with 1200 µm (4% of the cross-sectional area) and 2400 µm (16% of the cross-sectional area) diameter pores resulted in a flattening of the surface along the edge where Lode angle parameter equals 1, which is where the three notched tension tests lie, as shown in Figure 6b. The maximum stress model captured the notched tension tests well, but all surfaces predict much higher stress to failure than observed for the pure shear test. However, it should be noted that experimentally measured failure under shear should be taken as a lower bound (see, e.g., Ref. [27]). In general, the models capture the data well in equivalent stress spaces for the 316L, however the limited loss in strength with increased pore size makes it challenging to differentiate the calibrated fracture surfaces from one another compared to the equivalent strain space.

#### 3.2.2. L-PBF Ti-6Al-4V

**Figure 7:**
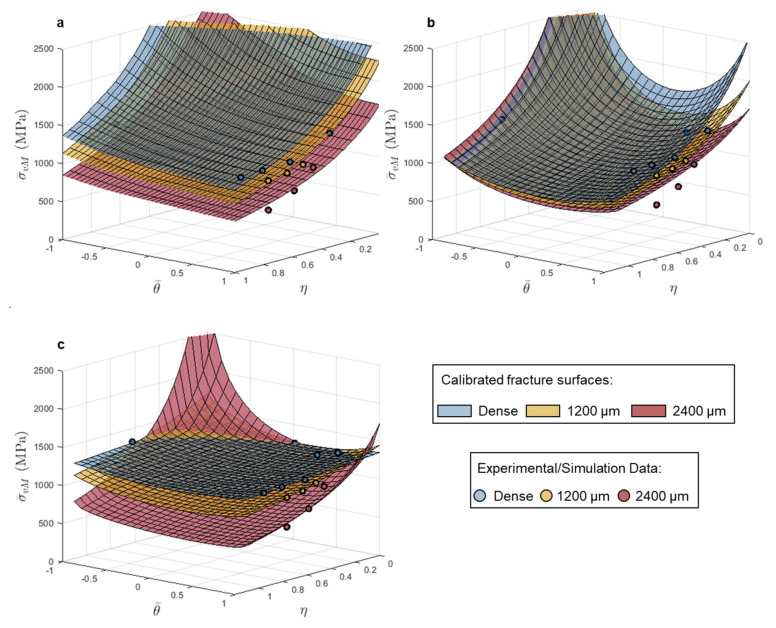
Calibrated (**a**) maximum stress, (**b**) Mohr–Coulomb, and (**c**) Hosford–Coulomb fracture loci in stress triaxiality vs. Lode angle parameter vs. equivalent stress space for Ti-6Al-4V using dense, 1200 µm pore, and 2400 µm pore data.

As the stress-based fracture models investigated here were developed to describe fracture behavior of brittle materials, it follows that a stress space criterion would better capture the detriment to mechanical behavior, due to the introduction of large pores, in already limited-ductility Ti-6Al-4V, better than a strain space representation based on plastic deformation. For Ti-6Al-4V, there were larger differences in the two models’ abilities to capture failure behavior with increased pore size; the MC model average MAPE was 9%, and the model had the most difficulty capturing the dense behavior (13.4% MAPE), but for the HC model the average MAPE was only 3%. The maximum stress model was able to capture the drop in equivalent stress to failure for samples with increased pore size; however, it did a poor job capturing the pure shear and punch tests, as shown in Figure 7a. 

Between the MC and HC models, the most evident change in calibrated fracture surface shape in stress space was between the surfaces for samples with 1200 µm diameter (4% of the cross-sectional area) and 2400 µm diameter (16% of the cross-sectional area) pores, as shown in Figure 7c. The calibrated HC surfaces showed a clear change from relatively stress state independent failure (flat surface) to more stress state dependent failure in the equivalent stress space for samples with the 2400 µm diameter (16% of the cross-sectional area) pore. For samples with a pore diameter of 1200 µm (4% of the cross-sectional area), the contributions of the elastic and plastic strain components were similar in magnitude; at failure the equivalent plastic strain was less than 1.5% for all three notched tension geometries. All samples with a pore diameter of 2400 µm (16% of the cross-sectional area), failed in an elastic deformation-dominated regime, for which there was significantly greater resolution in stress compared to plastic strain. Therefore, the brittle fracture derived models, based on strength limits, better captured the fracture behavior with the 1200 µm (4% of the cross-sectional area) and the 2400 µm (16% of the cross-sectional area) diameter pores.

## 4. Conclusions

This study takes advantage of the layer-by-layer manufacturing capabilities of AM to study the impact of controlled internal pores in two different metal alloys on stress-state and flaw-size dependent failure behavior. Fracture models presented in both equivalent stress (versus stress state) and strain (versus stress state) space were calibrated and their efficacy for describing the experimental/simulation results were discussed. The primary conclusions of this study are:

- L-PBF 316L and Ti-6Al-4V were shown to have drastically different stress state dependent fracture behavior in the dense condition, and these differences were exacerbated with the introduction of internal pores. Ultimately, the fracture behavior of relatively high ductility, and therefore defect tolerant, 316L was better captured by ductile fracture models based on an accumulation of damage with plastic deformation due to the significant plastic deformation to fracture observed in all samples, including those with pores. Conversely, the fracture behavior of relatively low ductility, and defect intolerant, Ti-6Al-4V was better captured by the fracture models derived based on critical strength values due to the limited or negligible plastic deformation preceding failure, particularly in samples with pores.
- For L-PBF 316L, the inclusion of the 1200 µm (4% of the cross-sectional area) and the 2400 µm (16% of the cross-sectional area) diameter pores in samples resulted in calibrated fracture surfaces in equivalent plastic strain space that had reduced stress state dependent failure, or flatter fracture surfaces, with increased pore size as failure in these samples became dominated by pore size rather than stress triaxiality.
- The effect of pores on the fracture behavior of L-PBF 316L was best captured in equivalent plastic strain space as significant equivalent plastic strain to failure was retained even with the samples that had the largest diameter pores (2400 µm or 16% of the cross-sectional area). Specifically, the modified Mohr-Coulomb model calibrated with pure shear, equibiaxial tension, and three unique round notched tension geometries (with and without intentional penny-shaped pores of varying diameters) most accurately captured the failure behavior of L-PBF 316L.
- For L-PBF Ti-6Al-4V, the use of equivalent stress-based fracture models, initially proposed for brittle materials, to evaluate the effect of internal pores of varying diameter was shown to be most appropriate.
- The equivalent stress-based Hosford-Coulomb failure criterion most accurately captured the failure behavior of L-PBF Ti-6Al-4V samples as a function of pore size. For samples with the largest diameter pores (2400 µm or 16% of the cross-sectional area), the fracture behavior, as visualized with the HC fracture surfaces in stress space, became more stress state dependent compared to the calibrated model for dense Ti-6Al-4V.

## Figures and Tables

**Table 1:** Calibrated stress-based fracture model parameters for 316L using data from dense pure shear and equibiaxial tension tests combined with dense, 300 µm, 600 µm, 1200 µm, and 2400 µm pore round notched tension tests. Error is the mean absolute percentage error for the model predicted values of equivalent stress on the fracture surface compared to the experimental values of equivalent stress across each stress state used in calibration.

L-PBF 316L		Pore Diameter (µm)				
		Dense	300	600	1200	2400
**Max Stress**	$\sigma_{max}$	1135	1151	1128	1073	1017
**Mohr-Coulomb**	$c_{1}$	0.516	0.507	0.468	0.383	0.337
	$c_{2}$	800	788	736	624	564
	**Error**	5.2%	5.0%	5.6%	6.7%	9.7%
**Hosford-Coulomb**	**a**	1.11	1.32	1.42	1.44	1.50
	**b**	1519	1452	1431	1427	1414
	**c**	0.422	0.392	0.414	0.451	0.500
	**Error**	4.9%	4.6%	5.0%	6.3%	8.7%

**Table 2:** Calibrated stress-based fracture model parameters for Ti-6Al-4V using data from dense pure shear and equibiaxial tension tests combined with dense, 300 µm, 600 µm, 1200 µm, and 2400 µm pore round notched tension tests. Error is the mean absolute percentage error for the model predicted values of equivalent stress on the fracture surface compared to the experimental values of equivalent stress across each stress state used in calibration.

L-PBF Ti-6Al-4V		Pore Diameter (µm)				
		Dense	300	600	1200	2400
**Max Stress**	$\sigma_{max}$	1817	1774.33	1647	1507	1128
**Mohr-Coulomb**	$c_{1}$	0.959	0.956	0.760	0.675	0.521
	$c_{2}$	1456	1429	1150	1030	814
	**Error**	13.4%	9.9%	7.5%	4.7%	9.6%
**Hosford-Coulomb**	**a**	1.23	0.944	1.06	0.862	0.438
	**b**	1376	1498	1439	1557	2428
	**c**	0.055	0.133	0.133	0.230	1.20
	**Error**	1.2%	1.2%	1.7%	2.3%	9.6%

**Table 3:** Calibrated strain-based ductile fracture model parameters for 316L using data from dense pure shear and equibiaxial tension tests combined with dense, 300 µm, 600 µm, 1200 µm, and 2400 µm pore round notched tension Table 1. which represents perfect model agreement with experiments.

L-PBF 316L		Pore Diameter (µm)				
		Dense	300	600	1200	2400
**Modified Mohr-Coulomb**	$c_{1}$	0.724	0.627	0.672	0.804	1.026
	$c_{2}$	1665	1292	1256	1225	1190
	$c_{\theta }^{s}$	1.99	1.61	1.53	1.41	1.23
	$c_{\theta }^{c}$	0.995	0.981	0.917	0.8	0.623
	**Error**	5.8%	4.3%	1.7%	4.3%	7.3%
**Hosford-Coulomb**	**a**	0.551	0.562	0.705	1.04	1.16
	**b**	1.34	1.34	1.09	0.688	0.553
	**c**	0.274	0.32	0.249	0.24	0.267
	**d**	0.473	0.352	0.309	0.377	0.321
	**Error**	7.1%	3.2%	0.7%	5.5%	17%

**Table 4:** Calibrated strain-based ductile fracture model parameters for Ti-6Al-4V using data from dense pure shear and equibiaxial tension tests combined with dense, 300 µm, 600 µm, 1200 µm, and 2400 µm pore round notched tension tests. Error is mean absolute percentage error for the model-calibrated damage accumulation in all five tests compared to a damage value of 1, which represents perfect model agreement with experiments.

L-PBF Ti-6Al-4V		Pore Diameter (µm)				
		Dense	300	600	1200	2400
**Modified Mohr-Coulomb**	$c_{1}$	0.069	0.109	0.139	0.156	0.219
	$c_{2}$	694	697	697	697	690
	$c_{\theta }^{s}$	0.981	0.982	0.978	0.976	0.954
	$c_{\theta }^{c}$	1.037	0.995	0.961	0.944	0.87
	**Error**	1.3%	11%	3.3%	32%	40%
**Hosford-Coulomb**	**a**	0.46	0.638	1.22	1.33	1.33
	**b**	0.451	0.478	0.193	0.236	0.235
	**c**	0.415	0.296	0.075	0.058	0.057
	**d**	0.154	0.059	0.018	0.008	0.008
	**Error**	3.7%	1.3%	8.4%	17%	58%

## Data Availability

Data available on request. The data presented in this study are available on request from the corresponding author.
